# Validation and applicability of the Tampa Difficulty Score for assessing procedural complexity in robotic liver surgery

**DOI:** 10.1007/s00464-025-12507-5

**Published:** 2026-02-23

**Authors:** Alexander Wilk, Thorsten Brechmann, Benno Mann

**Affiliations:** 1 Ruhr-Universität Bochum, Bochum, Germany; 2 Augusta-Klinikum Bochum Mitte, Clinic for General, Visceral and Robotic Surgery, Augusta-Kranken-Anstalt gGmbH, Bochum-Mitte, Bergstraße 26, 44791 Bochum, Germany; 3 Department of Gastroenterology and Oncology, Knappschaft Kliniken Bottrop, Bottrop, Germany

**Keywords:** Robotic liver surgery, Hepatobiliary surgery, Minimal invasive liver surgery, Oncologic surgery, Difficulty scoring system

## Abstract

**Background:**

With the increasing adoption of robotic systems in hepatic surgery, standardized assessment of procedural difficulty has become crucial, particularly for case selection in training and education. The Tampa Difficulty Score (TDS) was developed to classify robotic liver resections into four levels of complexity. This study aimed to externally validate the TDS.

**Methods:**

Seventy-nine consecutive patients undergoing robotic liver resection between 2018 and 2024 were included in this retrospective single-center study. Group comparisons between TDS categories were performed using descriptive and inferential statistics including analysis of variance via the Kruskal–Wallis test, Chi-square test, Mann–Whitney-*U* test, or Fisher’s exact test, as appropriate. Effect sizes were calculated accordingly. Post hoc analyses for intergrupal differences were conducted using the Kruskal–Wallis test. Spearman’s rank correlation assessed linear associations between TDS and perioperative variables.

**Results:**

Significant intergroup differences between TDS categories were found for major resections (p < 0.001), operative time (p < 0.001), blood loss (p < 0.001), intensive care stay (p = 0.002), hospital stay (p = 0.005), specimen weight (p < 0.001) and tumor size (p = 0.012). Post hoc analyses with Bonferroni–Holm correction confirmed significant differences mainly between TDS groups 2 and 3 for key parameters, but also showed homogenous distribution throughout the other TDS groups. Strong positive correlations were observed between TDS and major resections (|ρ| = 0.651), operative time (|ρ| = 0.715), blood loss (|ρ| = 0.507), and specimen weight (|ρ| = 0.578; all p < 0.001).

**Conclusion:**

The Tampa Difficulty Score was validated for RLR, showing consistent results in both intergroup comparisons and correlation analyses. TDS proved to be a reliable instrument for assessing procedural difficulty, demonstrating its value for risk assessment, training standardization, as well as future evaluations despite the limited sample size.

**Graphic Abstract:**

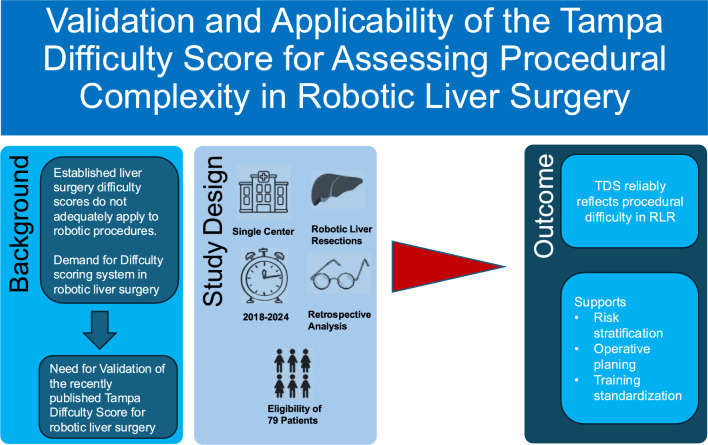

**Supplementary Information:**

The online version contains supplementary material available at 10.1007/s00464-025-12507-5.

The field of liver surgery has been historically dominated by the open approach (open liver resection, OLR), primarily due to the complex hepatic anatomy and the superior visualization of the operative field it provided [1]. With the advent of the minimal invasive liver surgery (MILS) era this paradigm gradually shifted toward less invasive techniques. Laparoscopic liver resection (LLR) demonstrated non-inferior and regarding some parameters even superior, perioperative outcomes compared to open surgery, including reduced blood loss, comparable operative times, and shorter lengths of stay in both the intensive care unit (ICU) and the hospital overall [2, 3]. In high-volume tertiary centers with substantial expertise even complex hepatic procedures have proven to be feasible via LLR [4–6]. Nevertheless, inherent technical limitations of conventional laparoscopy, such as the use of rigid, manually controlled instruments and restricted degrees of freedom have limited the widespread adoption of LLR beyond minor hepatic resections and outside of specialized expert centers [7–11].

Technological advancements within the field of MILS embodied in the use of robotic liver resection (RLR), representing a true integration of complex hepatic surgery with a minimal invasive approach. Features such as tremor filtration, enhanced dexterity through articulating instruments and high-definition three-dimensional visualization have expanded the indications for minimal invasive procedures while maintaining oncological radicality comparable to OLR or LLR. Moreover, RLR appears to offer a shorter learning curve than LLR, enabling surgeons to perform major hepatic resections earlier without compromising patient safety [12–18].

To allow for standardized preoperative assessment of procedural complexity and to aid aspiring surgeons through the learning curve, several difficulty scoring systems (DSS) have been proposed [19–25]. These scores have proven valuable for laparoscopic liver resections, incorporating parameters related to anatomical, technical and oncological factors. However, with the advent of RLR, enabling major anatomical resections and technically demanding biliary or vascular reconstructions, the existing laparoscopic DSS no longer reflect the procedural challenges specific to robotic approaches adequately and are therefore not directly applicable to RLR.

Sucandy et al. introduced a novel DSS specifically tailored to robotic liver resections, addressing the evolving requirements of this technique [26]. The Tampa Difficulty Score (TDS) comprises seven parameters that consider oncological, anatomical, and procedure-specific considerations. In detail those parameters include neoadjuvant chemotherapy, tumor location, tumor size, tumor type, extent of parenchymal resection, the need for portal lymphadenectomy and the requirement for hepatobiliary reconstruction with hepaticojejunostomy following extrahepatic biliary resection. Each category is assigned a defined score, resulting in a cumulative value ranging from 1 to 49 points. Patients are subsequently stratified into four difficulty groups, with group 4 representing the most complex surgical procedures.

The aim of this study was to evaluate the applicability of the TDS for robotic liver surgery and to investigate its association with perioperative outcomes, thereby enabling valid complexity stratification for robotic hepatic procedures.

## Methods

### Study population

The institutional liver surgery database was analyzed in a single-center retrospective study, including consecutive adult patients who underwent liver resection at Augusta Academic Hospital Bochum, Germany, between January 2010 and October 2024. In total, 202 patients were identified. Of these, all OLR and LLR were excluded, yielding 99 patients who underwent RLR. Following exclusion of patients who underwent concomitant procedures involving other organs, a total of 79 patients remained eligible for inclusion. All RLRs were performed between January 2018 and October 2024. The extent and type of hepatic resection were determined by a multidisciplinary tumor board, considering tumor location, pathology, and the overall clinical condition of each patient. Comparative perioperative data on OLR and RLR derived from this database have been reported previously [27]. In the present study, additional parameters were analyzed and evaluated in relation to a distinct hypothesis, as defined by the primary and secondary endpoints.

### Data collection

Data were collected from patient records in the clinic’s database. Demographic characteristics included age, sex, body mass index (BMI), ASA classification, prior surgeries, chemotherapy history, liver diseases, preoperative laboratory values, and the indication for surgery. Characteristics of the surgical procedure included the extent of parenchymal resection, need for portal lymphadenectomy, need for extrahepatic biliary resection with hepaticojejunal reconstruction, intraoperative complications, conversion rate, precisely measured blood loss (BL), blood transfusions, peridural catheter placement, duration of the operation and the devices used during surgery. Postoperative parameters included the length of stay in the intensive care unit and the hospital, complication rates according to the Clavien-Dindo classification, reoperation rate, and postoperative laboratory values. Histopathological analysis encompassed the entity and the R-status in the cases of a benign or malignant tumour, liver weight, tumor location and tumor size.

### Score calculation

The TDS for each patient was determined based on a point-based assessment encompassing seven parameters: neoadjuvant chemotherapy, tumor location, tumor size, tumor type, extent of parenchymal resection, need for portal lymphadenectomy, and requirement for hepatobiliary reconstruction with hepaticojejunostomy following extrahepatic biliary resection. Each patient was subsequently assigned to one of the four TDS categories.

### Primary and secondary objectives

The primary objective was testing for significant differences across the four TDS categories and relevant perioperative parameters.

Secondary endpoints included post-hoc analysis of significant differences as well as the evaluation of linear correlations between the TDS groups and relevant perioperative or histopathological parameters.

### Statistical analysis

The study population was divided into four groups based on the calculated TDS A statistical comparison between the TDS groups was conducted for demographic data as well as primary and secondary endpoints. Statistical methods included descriptive statistics with median and interquartile ranges (IQR) as well as interferential statistics using non-parametric tests including the Kruskal–Wallis test, chi-square test, Mann–Whitney *U* test and Fisher’s exact test. Post-hoc analysis was conducted if the Kruskal-Wallis test revealed significant differences between the TDS groups or for key parameters using Bonferroni-Holm-corrected Kruskal–Wallis tests for independent samples. A *p*-value of ≤ .05 was regarded as statistically significant and denoted in boldface in the tables. Additionally, linear correlation between the TDS and key parameters were assessed using Spearman’s rank correlation. Parameters included in the correlation analysis comprised key variables of the TDS as well as parameters showing significant differences between the TDS groups in the Kurskal–Wallis test. Correlation coefficients were defined as weak (|ρ| = 0.10), moderate (|ρ| = 0.30), and strong (|ρ| = 0.50) correlations [28]. Statistics were realized with SPSS version 29.0.2.0 (20) (IBM SPSS Statistics License: IBM SPSS Statistics). Effect sizes were calculated online [29]. Measures of effect size were defined as weak (d ≥ 0.2), moderate (d < 0.5) and strong (d ≥ 0.8) [28]. The authors possessed proficiency in statistical methodology. All statistical analyses were conducted by Brechmann and Wilk.

### Definitions

The classification of liver segments followed the standardized Couinaud classification [30]. Major liver resections were defined as the removal of at least three segments. In cases of partial segmental resections, the segment count was rounded down to the lower number. The operation time was defined as the period between the skin incision and the completion of skin suture. Blood loss (BL) was determined as the precisley measured volume within the suction device at the end of the operation. A conversion during robotic surgery was defined as any laparotomy performed for reasons other than specimen retrieval. Further distinctions were made between emergency and planned conversions. Planned conversions were performed for patients deemed unsuitable for robotic surgery, while emergency conversions were necessitated by time constraints in combination with life-threatening conditions. Intraoperative complications were classified as minor or major. Major complications included injuries of large blood vessels, like the portal vein, hepatic veins, inferior Vena Cava or arterial injuries. Minor complications included bowel injuries, bile duct sutures, or minor bleeding. Postoperative complications were classified according to Clavien-Dindo with major complications being those classified as grade 3 or higher [31]. Histological resection status was defined according to the TNM classification [32]. The reported resected tissue weight referred to liver tissue only.

### Operation procedures

All procedures were performed by an experienced hepatobiliary surgeon. The robotic procedures were conducted using the Da Vinci X system in the supine position with 10–15 degrees of Anti-Trendelenburg and a slight left tilt. Capnoperitoneum was established via Palmer's point. Four 8 mm robotic ports were inserted in a curved arrangement approximately 10–15 cm from the target anatomy, with at least 8 cm spacing between them. Additionally, one or two assistant ports (12 mm and 5 mm) were placed. After ruling out contraindications for surgical resection, the liver was mobilized. The portal triad within the hepatoduodenal ligament was identified and ligated using Hemo-o-Lock clips. Parenchymal transection was performed utilizing bipolar forceps, a water jet device and/or a vessel-sealing device. At the conclusion of this phase, the corresponding hepatic vein was resected using a robotic stapler with a white cartridge. The specimen was extracted through a Pfannenstiel-like incision. Routine placement of a drain was not performed.

### Ethical approval

The study protocol was approved by the institutional review board of Ruhr University Bochum (registry number DE/EKNW34) in accordance with the ethical guidelines of the Declaration of Helsinki and its subsequent revisions. Informed consent was obtained from all patients prior to undergoing surgery. Due to the retrospective design, the requirement for informed consent was waived by the institutional review board.

### Drafting the manuscript

The manuscript was initially drafted in English. To enhance readability, generative artificial intelligence (ChatGPT) was utilized for rephrasing the text. Subsequently, the entire manuscript was meticulously proofread and reviewed by the authors. It should be noted that no content was originally analyzed, organized or conceptualized by the generative AI. The STROBE statement was used for reporting guidelines.

## Results

### Demographic characteristics

A total of 79 patients (female: 37 (46.8%)) were included, with 3 patients in the first Tampa group, 42 patients in the second Tampa group, 31 patients in the third Tampa group and 3 patients in the fourth Tampa group. The four groups were comparable with respect to age, gender, body mass index (BMI), American Society of Anesthesiologists (ASA) score, preoperative laboratory values and pre-existing liver disease. Significant differences were observed regarding the history of prior abdominal surgery (p = 0.001), prior hepatic surgery (p = 0.041), prior chemotherapy (p < 0.001) and surgical indication (p < 0.001) (Tables 1 and 1-S).

**Table 1:** Demographic characteristics

	Valid cases	Total Cohort n=79 Median [IQR] or number (%)*	Tampa group 1 n=3 Median [IQR] or number (%)*	Tampa group 2 n=42 Median [IQR] or number (%)*	Tampa group 3 n=31 Median [IQR] or number (%)*	Tampa group 4 n=3 Median [IQR] or number (%)*	*p*-value^A^
Florida Difficulty Index			1–8	9–24	25–32	33–49	
Tampa Score	79	24 [12; 36]	8	17 [13; 21]	27 [26; 29]	39	
Age [years]	79	63 [58; 73]	71 [45; 71]	68 [58; 76]	63 [57; 70]	59 [58; 59]	.431
Gender [female]	79	37 (46.8)	2 (66.7)	19 (45.2)	16 (51.6)	0 (0)	.328
BMI [kg m^−2]^	78	25 [23; 29]	21 [19; 21]	25 [23; 29]	24 [23; 31]	28 [26; 28]	.217
ASA Score ^+^	79	3 {2.61 ± 0.6}	3 {2.67 ± 0.6}	3 {2.55 ± 0.6}	3 {2.68 ± 0.6}	3 {2.67 ± 0.6}	.901
ASA Scoring							.901
1		1 (1.3)	0 (0)	0 (0)	1 (3.2)	0 (0)	
2		31 (39.2)	1 (33.3)	20 (47.6)	9 (29.0)	1 (33.3)	
3		45 (57.0)	2 (66.7)	21 (50.0)	20 (64.5)	2 (66.7)	
4		2 (2.5)	0 (0)	1 (2.4)	1 (3.2)	0 (0)	
History of prior surgery	79						
Abdominal		61 (77.2)	0 (0)	30 (71.4)	29 (93.5)	2 (66.7)	**.001**
Hepatic		11 (13.9)	0 (0)	4 (9.5)	5 (16.1)	2 (66.7)	**.041**
History of prior chemotherapy	79	38 (48.1)	0 (0)	13 (31.0)	24 (77.4)	1 (33.3)	**< .001**
Pre-existing liver disease	79						.950
Hepatic steatosis		2 (2.5)	0 (0)	1 (2.4)	1 (3.2)	0 (0)	
Liver cirrhosis		7 (8.9)	0 (0)	4 (9.5)	2 (6.5)	1 (33.3)	
Viral hepatitis		3 (3.8)	0 (0)	2 (4.8)	1 (3.2)	0 (0)	

^*^As appropriate

^+^ presented as Median {mean ± SD}

^A^ Statistics were realised by Fisher’s exact test, Chi^2^ test, Man–Whitney *U*-test or Kruskal–Wallis-test, as appropriate

### Characteristics of the surgical procedures

In the total cohort, 38 patients (48.1%) underwent a major resection. The median operating room time (OR time) was 202 minutes [IQR 136; 264]. Median BL was 200 milliliters [IQR 50; 500]. Furthermore, no significant differences were observed regarding the TDS groups and intraoperative complications, conversion rates, transfusion rates, peridural catheter placement, sealing or dissection devices. The Kruskal-Wallis test revealed statistically significant differences between the Tampa Score groups and major resections (p < 0.001), OR time (p < 0.001), blood loss (p < 0.001) and the use of stapling devices (p = 0.003). The post-hoc analysis with Bonferroni-Holm correction for major resections showed statistically significant differences between group 1 and group 3 (p = 0.035; d = .899) and between group 2 and group 3 (p < 0.001; d = 1.55). For OR time, statistically significant differences were found between group 1 and group 3 (p = 0.001; d = 1.635), group 1 and group 4 (p = 0.001; d = n/a), group 2 and group 3 (p < 0.001; d = 1.422) and group 2 and group 4 (p = 0.002; d = 1.272) with large effects. For BL group 1 and group 3 (p = 0.003; d = 1.481), group 1 and group 4 (p = 0.011; d = n/a) and group 2 and group 3 (p = 0.005; d = 0.861) differed significantly with large effects (Table 2, and Tables 2-S, 3-S, 4-S, 5-S, 6-S).

**Table 2:** Outcomes based on the Tampa Diffculty Scoring System

Group	Valid cases	Tampa group 1 n=3 Median [IQR] or number (%)*	Tampa group 2 n=42 Median [IQR] or number (%)*	Tampa group 3 n=31 Median [IQR] or number (%)*	Tampa group 4 n=3 Median [IQR] or number (%)*	p-value^A^
Florida Difficulty Index		1–8	9–24	25–32	33–49	
Tampa Score	79	8	17 [13; 21]	27 [26; 29]	39	
Major resection	79	0 (0)	9 (21.4)	26 (83.9)	3 (100.0)	**< .001**
Intraoperative complication (major)	79	0 (0)	1 (2.4)	3 (0.7)	0 (0)	.499
OR time [min]	78	68 [54; 68]	171 [130; 202]	263 [234; 322]	420 [405; 420]	**< .001**
Blood loss [ml]	79	10 [10; 10]	100 [50; 200]	300 [200; 600]	500 [200; 500]	**< .001**
Emergency conversion rate	79	0 (0)	0 (0)	1 (3.2)	0 (0)	.873
Length of stay	79					
ICU [d]		0 [0; 0]	0 [0; 1]	1 [1; 2]	1 [1; 1]	**.002**
In total [d]		2 [2; 2]	8 [6; 12]	11 [8; 18]	15 [7; 15]	**.005**
Major complicaiton^a^	79	0 (0)	10 (23.8)	8 (25.8)	1 (33.3)	.637
Liver specific complications^b^	79	0 (0)	6 (14.3)	7 (22.6)	1 (33.3)	.833
Re-operation	78	0 (0)	6 (14.6)	3 (9.7)	1 (33.3)	.580
Histologic outcome						
R0	77	3 (100)	37 (90.2)	26 (86.7)	3 (100)	.803
Weight of resected liver tissue [g]	77	79 [25; 79]	180 [44; 310]	740 [432; 106]	514 [180; 514]	**< .001**
Tumor size [cm]	79	2.4 [2.3; 2.4]	2.9 [2.3; 5.0]	4.5 [3.6; 5.8]	4.8 [2.7; 4.8]	**.012**

^*^As appropriate

^+^ presented as Median {mean ± SD}

^A^ Statistics were realised by Fisher’s exact test, Chi^2^ test, Man–Whitney *U*-Test or Kruskal–Wallis–test, as appropriate

^a^ According to Clavien–Dindo–Complication-Score

^b^ Ascites, Liver–failure, Perihepatic fluid collection/abscess, Bile leakage

### Postoperative characteristics

In the total cohort, the median LOS in the ICU was 1 day [IQR 0; 1] and the total hospital stay was 9 days [IQR 7; 15]. Nineteen patients (24.1%) experienced a minor postoperative adverse event, while 19 patients (24.1%) had a major adverse event with no statistical significant difference within the groups (p = 0.637). According to the Clavien–Dindo classification, Grade II complications represented the most common category in the overall cohort, occurring in 20.3% of patients (n = 16). There was a notable, yet not statistically significant trend in the occurrence rates of liver-specific complications. As for liver-specific complications the trend showed as follows: group 2 (n = 6; 14.3%) vs. group 3 (n = 7; 22.6%) vs. group 4 (n = 1; 33.3%) (p = 0.833). In total, 10 patients (12.7%) required reoperation without showing significant differences between the Tampa Score groups (p = 0.580). Overall death occurred in 4 patients (5.1%), of which 3 patients deceased postoperatively. Statistically significant differences between the Tampa Score groups were observed regarding LOS on the ICU (p = 0.002) and total hospital stay (p = 0.005). No significant differences were observed regarding postoperative adverse events (p = 0.637). Concerning postoperative laboratory parameters, significant differences were found in CRP (p = 0.037), bilirubin (p = 0.002), ALT (p < 0.001), AST (p < 0.001), GGT (p < 0.001), AP (p < 0.001), LDH (p = 0.003) and lactate (p = 0.002) between the TDS groups. The post-hoc analysis for ICU stay revealed statistically significant differences only between group 2 and group 3 (p = 0.011; d_cohen_ = 0.782). For total hospital stay, statistically significant differences were found between group 1 and group 3 (p = 0.024; d_cohen_ = 1.131) (Table 2, Tables 7-S, 8-S, 9-S, 10-S, 11-S).

### Histopathological results

Colorectal metastases represented the most common histopathological entity, accounting for 48.1% of cases (n = 38). The second most frequent entity were primary liver tumors, accounting for 22.8% of cases (n = 18). A complete (R0) resection was achieved in 89.6% of all cases, with no significant differences between the groups (p = 0.803). The median weight of resected liver tissue was 292 g [IQR 82; 734] and the tumor size was 4.1 cm [IQR 2.4; 4.2]. Histopathological entities differed significantly between the TDS groups (p = 0.001). Within the Tampa Score groups, significant differences were observed for resected liver weight (p < 0.001) and tumor size (p = 0.012). The post-hoc analysis for resected liver weight showed statistically significant differences between group 1 and group 3 (p = 0.027; d_cohen_ = 1.115), group 1 and group 4 (p = 0.280; d_cohen_ = 2.746) and group 2 and group 3 (p < 0.001; d_cohen_ = 1.313) with large effect sizes. For tumor size a difference with moderate effect was found between group 2 and group 3 (p = 0.029; d_cohen_ = .698) (Table 2, Tables 12-S, 13-S, 14-S).

### Correlation analysis

**Fig. 1:**
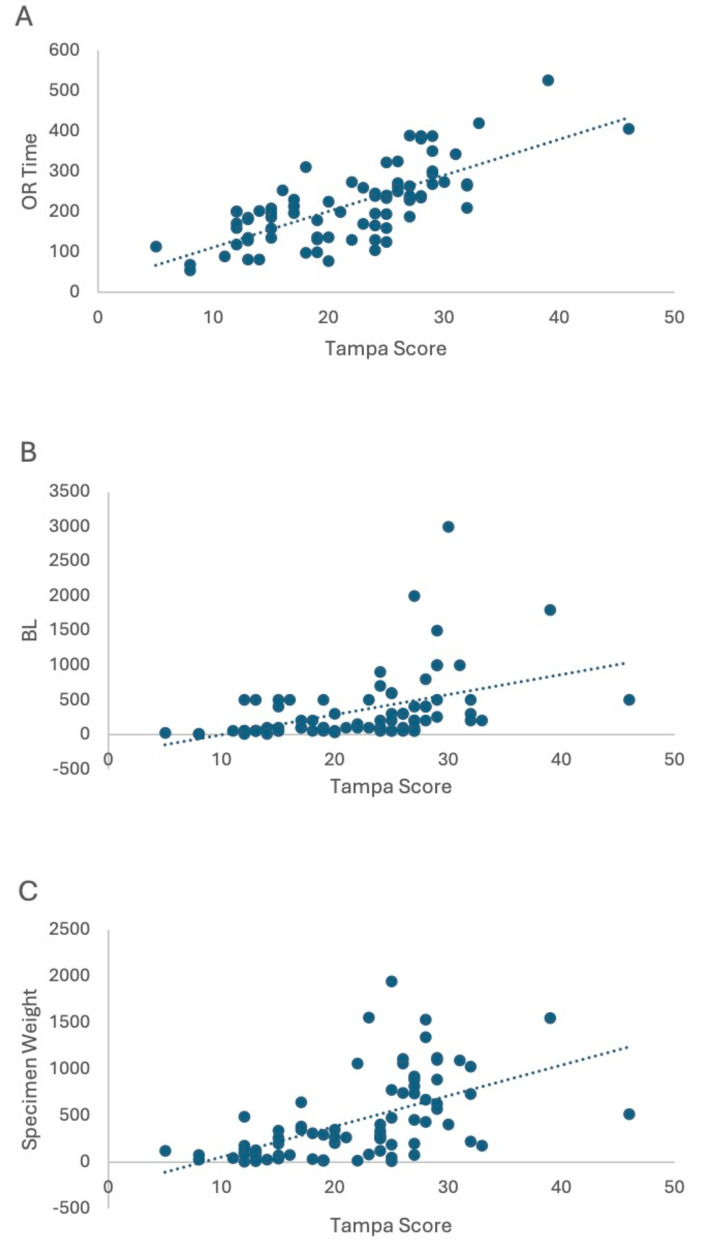
**A** Correlation between OR-Time and the TDS (|rho| = 0.715; p < 0.001) **B** Correlation between BL and the TDS (|rho| = 0.507 p < 0.001) **C** Correlation between specimen weight and der TDS (|rho| = 0.578; p < 0.001)

Strong positive linear correlations were identified between the Tampa Difficulty Score and the following parameters: proportion of major resections (|ρ| = 0.651; p < 0.001), operative time (|ρ| = 0.715; p < 0.001), blood loss (|ρ| = 0.507; p < 0.001) and specimen weight (|ρ| = .578; p < 0.001). Moderate positive linear correlations were observed for tumor size (|ρ| = 0.365; p < 0.001), LOS in the ICU (|ρ| = 0.437; p < 0.001) and total hospital stay (|ρ| = 0.382; p < 0.001). TDS score correlated significantly and positive with the number of intraoperative complications with a small effect size (|ρ| = .294; p = 0.009). No significant linear correlation could be observed for the onset of postoperative complications (|ρ| = 0.166; p = 0.143) (Table 3; Fig. 1 ).

**Table 3:** Tampa groups correlations

	Valid cases	Tampa groups correlation coefficient (|ρ|)^A,B^	*p*-value^A^
Minor/major resection	79	**.651**	<0.001
OR-time	78	**.715**	<0.001
BL	79	**.507**	<0.001
Intraoperative complications	79	.294	.009
Liver weight	79	**.578**	<0.001
Tumor size	79	.365	<0.001
Postoperative minor/major complications^a^	79	.166	.143
ICU stay	79	.437	<0.001
Hospital stay	79	.382	<0.001

^a^ According to clavien-dindo-complication-score

^A^ Statistics were realised by Spearman`Rho

^B^ weak correlation (|ρ| ≥ 0.10), moderate correlation (|ρ| ≥ 0.30), strong correlation (|ρ| ≥ 0.50) displayed as bold

## Discussion

We were able to demonstrate, through various statistical approaches including analysis of variance with post hoc testing as well as correlation analysis, that the Tampa Difficulty Score represents a valuable instrument for discriminating and classifying robotic liver resections according to their complexity and difficulty. Not only were significant differences observed between the overall Tampa Score groups regarding relevant perioperative parameters, but in many cases the four groups also differed significantly in pairwise comparisons. This post hoc analyses demonstrated a homogeneous distribution and mostly strong effect sizes of intergroup differences across various TDS parameters, indicating that the observed significant results in the analysis of variance were not attributable to small sample size only, but rather to genuine differences between groups and parameters. Significant differences were further noted in postoperative inflammatory markers, transaminases and cholestatic parameters. As expected, those postoperative laboratory values were higher in the TDS groups with more extensive or complex procedures, reflecting the corresponding cellular stress response. Moreover, several tested perioperative variables exhibited meaningful moderate to high correlations with the TDS, meaning, that those parameters were increasing with higher TDS and in reverse. However, it should be emphasized that this merely reflects simultaneous variation between parameters and must not be interpreted as causality. Interestingly, no significant differences were detected with respect to the occurrence of adverse events. The incidence of major postoperative complications, approximately 25%, was already evident in the second TDS group but remained comparable across higher complexity levels. Liver-specific complications showed an increasing trend without reaching statistical significance. Likewise, intraoperative complications did not differ significantly between groups, though a weak linear correlation with TDS was observed. A potential explanation for the lack of significant differences or strong correlations in complication rates may lie in the inherently high morbidity of hepatic surgery; however, when combined with an experienced surgeon, this may have mitigated the incidence of major complications in more complex procedures.

As outlined earlier, several valuable difficulty scoring systems have been proposed, validated, and applied in LLR, incorporating procedure-specific factors for difficulty stratification. These scores have been shown to predict key perioperative parameters and short-term outcomes with varying accuracy depending on the model [22–25]. To some extent, these scores can be applied to RLR; however, none were specifically designed for RLR. To confirm the validity and clinical applicability of the TDS as a predictive instrument, particularly for surgeons in the early phases of their robotic learning curve, the score must undergo not only internal validation [33] but also external validation across broader patient populations and institutions. Al Harakeh et al. demonstrated the external applicability of the TDS in a validation cohort of 147 patients. Relevant parameters showing significant differences included the Child–Pugh score, OR-Time, BL, tumor size, proportion of major resections, postoperative complications and LOS-parameters [34]. These findings are largely consistent with our own results, supporting the general applicability of the TDS in this context. Additionally, Muttillo et al. recently demonstrated in a multicenter, retrospective, propensity score matched analysis of 218 eligible patients per study arm undergoing robotic liver resection that procedures involving tumors situated adjacent to major vascular structures constituted an additional level of technical difficulty, yet this circumstance was effectively manageable robotically and did not translate into increased postoperative morbidity.

Although other scoring systems, such as the IWATE or IMM, were able to predict intraoperative complications and unplanned conversions with reasonable accuracy, their overall performance across all evaluated metrics remained suboptimal, stressing the need for a more suitable and comprehensive scoring concept [35]. These findings highlight the clinical relevance, broader applicability and value of the TDS, particularly as it captures the multifactorial determinants of procedural complexity in RLR. Among other factors, the TDS accounts for tumor localisation in anatomically demanding areas such as posterior liver segments, malignant tumor entities, and the requirement for biliary resection with subsequent reconstruction. Moreover, the TDS may support perioperative planning by enabling a structured, reproducible assessment of procedural difficulty. The score allows surgeons to anticipate technical challenges more accurately while facilitating an appropriate allocation of operative resources. Beyond its practical utility in operative planning, the TDS also serves as a valuable educational instrument for aspiring hepatic surgeons. It may facilitate evidence-based case selection that corresponds to the surgeon’s level of experience, thereby promoting a stepwise progression along the learning curve and ultimately contributing to safer and a more effective training pathway.

Despite the high proportion of complex hepatic procedures and the overall good quality of the underlying patient data, several limitations must be acknowledged. Firstly, the investigated cohort is limited by its relatively small sample size of 79 procedures, resulting in unbalanced group sizes, particularly in TDS groups 1 and 4. This restricts the statistical power of the analyses, increases the risk of type II error, thereby reducing the robustness of comparisons across difficulty levels. Consequently, the reliability of effect estimates within these smaller categories is limited and observed trends should be interpreted with caution. However, it should be noted that procedures involving additional organ systems were excluded, thereby improving the homogeneity and overall quality of the analyzed dataset. Moreover, post hoc analyses were conducted with Bonferroni–Holm to minimize potential small-sample bias with significant results in the analysis of variance. Secondly, the present study is based on a retrospective cohort of RLR, inherently carrying the risk of information bias and incomplete data acquisition. Retrospective analyses depend on the accuracy and completeness of existing records, which may result in underreporting or inconsistencies. Thirdly, all procedures were performed by a single surgeon with extensive expertise in MILS and hepatic surgery. While this ensures a high degree of technical consistency, it inevitably limits the generalizability of the findings to institutions with lower procedural volumes or surgeons with less experience. The presented data should therefore be interpreted with appropriate circumspection. Whether the TDS is applicable to this subgroup remains uncertain and should be clarified by future studies specifically addressing this clinical context. Fourthly, no follow-up data was available for further analysis of long-term outcomes.

Regardless of these limitations, the present investigation provides a robust and well-characterized dataset on RLR, distinguished by a balanced demographic composition, inclusion of technically demanding procedures and a substantial proportion of major resections. These features contribute valuable evidence to the still limited body of data from Germany and Europe. Moreover, to the best of our knowledge, this study offers the first comprehensive analysis combining intergroup comparisons with testing for linear correlation in the context of TDS validation. Beyond mere statistical associations, our findings indicate that TDS holds practical significance for anticipating perioperative outcomes while supporting nuanced risk stratification. Integration of TDS into preoperative planning frameworks could therefore refine individualized surgical decision-making, guiding the choice of operative approach and resource allocation more effectively. We propose the probatory implementation of the TDS in tertiary centers for validation.

## Conclusion

This study provides one of the first external validations of the TDS, which was specifically developed for robotic liver resection. Through comprehensive statistical methodology, we demonstrated that the TDS allows for meaningful assessment of procedural complexity and risk stratification in robotic liver surgery, thereby filling an existing gap in preoperative evaluation tools. Further studies, preferably prospective, are warranted to include larger cohorts, a broader range of institutional experiences and a more balanced distribution across TDS groups in order to strengthen the existing body of evidence.

## Supplementary Information

Below is the link to the electronic supplementary material.

Supplementary file1 (DOCX 17 kb)

Supplementary file2 (DOCX 20 kb)

Supplementary file3 (DOCX 16 kb)

Supplementary file4 (DOCX 16 kb)

Supplementary file5 (DOCX 16 kb)

Supplementary file6 (DOCX 16 kb)

Supplementary file7 (DOCX 19 kb)

Supplementary file8 (DOCX 18 kb)

Supplementary file9 (DOCX 17 kb)

Supplementary file10 (DOCX 16 kb)

Supplementary file11 (DOCX 16 kb)

Supplementary file12 (DOCX 18 kb)

Supplementary file13 (DOCX 16 kb)

## Declarations

### Declaration of AI and AI-assisted technologies in the writing process

To enhance readability, generative artificial intelligence (ChatGPT) was utilised for rephrasing the text. It should be noted that no content was originally analysed, organised, or conceptualised by the generative AI.
