# Supplementary material for: Validation and applicability of the Tampa Difficulty Score for assessing procedural complexity in robotic liver surgery
Source: Surg Endosc. 2026 Feb 23;40(5):3852–62. doi: 10.1007/s00464-025-12507-5 (PMC13160962; doi:10.1007/s00464-025-12507-5)
Supplement: Supplementary file 1 — Supplementary file1 (DOCX 17 kb) [file 464_2025_12507_MOESM1_ESM.docx]

**Table 6-S:** Post-Hoc OR-Time

|  | **Tampa Group 1**  **n=3**  ***p*-value^A,B^** | **Tampa Group 2**  **n=42**  ***p*-value^A,B^** | **Tampa Group 3 n=31**  ***p*-value^A,B^** | **Tampa Group 4**  **n=3**  ***p*-value^A,B^** | **d_Cohen_** | **Eta squared (η2)** |
| --- | --- | --- | --- | --- | --- | --- |
| **Tampa Group 1**  **n=3**  ***p*-value^A,B^** | n/a | .468 | **.001** | **.001** | 1.635/n/a | 0.4/2.561 |
| **Tampa Group 2**  **n=42**  ***p*-value^A,B^** | .468 | n/a | **<.001** | **.002** | 1.422/1.272 | .336/.288 |
| **Tampa Group 3 n=31**  ***p*-value^A,B^** | **.001** | **<.001** | n/a | .659 | 1.636/1.422 | 0.4/.336 |
| **Tampa Group 4**  **n=3**  ***p*-value^A,B^** | **.001** | **.002** | .659 | n/a | n/a/1.272 | 2.561/.288 |
| ^A^ Statistics were realised by Independant-Samples Kruskal-Wallis-Test  ^B^ Significant Values have been adjusted by the Bonferroni-Correlation for multiple test | | | | | | |
