# Supplementary material for: Validation and applicability of the Tampa Difficulty Score for assessing procedural complexity in robotic liver surgery
Source: Surg Endosc. 2026 Feb 23;40(5):3852–62. doi: 10.1007/s00464-025-12507-5 (PMC13160962; doi:10.1007/s00464-025-12507-5)
Supplement: Supplementary file 2 — Supplementary file2 (DOCX 20 kb) [file 464_2025_12507_MOESM2_ESM.docx]

**Table 7-S:** Postoperative Characteristics

|  | **Valid cases** | **Total Cohort**  **n=79 Median [IQR] or number (%)*** | **Tampa Group 1**  **n=3**  Median [IQR] or number (%)* | **Tampa Group 2**  **n=42**  Median [IQR] or number (%)* | **Tampa Group 3 n=31**  Median [IQR] or number (%)* | **Tampa Group 4**  **n=3**  Median [IQR] or number (%)* | ***p*-value^A^** |
| --- | --- | --- | --- | --- | --- | --- | --- |
| **Length of stay** | 79 |  |  |  |  |  |  |
| ICU [d] |  | 1 [0; 1] | 0 [0; 0] | 0 [0; 1] | 1 [1; 2] | 1 [1; 1] | **.002** |
| In total [d] |  | 9 [7; 15] | 2 [2; 2] | 8 [6; 12] | 11 [8; 18] | 15 [7; 15] | **.005** |
| **Any complication^a^** | 79 |  |  |  |  |  | .637 |
| None |  | 41 (51.9) | 2 (66.7) | 25 (59.5) | 13 (41.9) | 1 (33.3) |  |
| Minor |  | 19 (24.1) | 1 (33.3) | 7 (16.7) | 10 (32.3) | 1 (33.3) |  |
| Major |  | 19 (24.1) | 0 (0) | 10 (23.8) | 8 (25.8) | 1 (33.3) |  |
| **Clavien-Dindo-complication-score** | 79 |  |  |  |  |  |  |
| I |  | 3 (3.8) |  |  |  |  |  |
| II |  | 16 (20.3) |  |  |  |  |  |
| IIIa |  | 6 (7.6) |  |  |  |  |  |
| IIIb |  | 6 (7.6) |  |  |  |  |  |
| IVa |  | 3 (3.8) |  |  |  |  |  |
| IVb |  | n/a |  |  |  |  |  |
| V |  | 4 (5.1) |  |  |  |  |  |
| **Re-Operation** | 78 | 10 (12.7) | 0 (0) | 6 (14.6) | 3 (9.7) | 1 (33.3) | .580 |
| ^a^ According to Clavien-Dindo-Complication-Score  ^A^ Statistics were realised by Fisher’s exact test, Chi^2^ test, Man-Whitney U-Test or Kruskal-Wallis-test, as appropriate | | | | | | | |
