# Supplementary material for: Validation and applicability of the Tampa Difficulty Score for assessing procedural complexity in robotic liver surgery
Source: Surg Endosc. 2026 Feb 23;40(5):3852–62. doi: 10.1007/s00464-025-12507-5 (PMC13160962; doi:10.1007/s00464-025-12507-5)
Supplement: Supplementary file 3 — Supplementary file3 (DOCX 16 kb) [file 464_2025_12507_MOESM3_ESM.docx]

**Table 8-S:** Surgical procedure related complications

|  | **Valid cases**  **n=XX** | **Total Cohort**  **n=79 Median [IQR] or number (%)*** | **Tampa Group 1**  **n=3**  Median [IQR] or number (%)* | **Tampa Group 2**  **n=42**  Median [IQR] or number (%)* | **Tampa Group 3 n=31**  Median [IQR] or number (%)* | **Tampa Group 4**  **n=3**  Median [IQR] or number (%)* | ***p*-value^A^** |
| --- | --- | --- | --- | --- | --- | --- | --- |
| **Liver Specific Complications** | 79 | 14 (17.7) | 0 (0) | 6 (14.3) | 7 (22.6) | 1 (33.3) | .833 |
| Ascites |  | 4 (5.1) |  |  |  |  |  |
| Liver-failure |  | 2 (2.5) |  |  |  |  |  |
| Perihepatic fluid collection / abscess |  | 1 (1.3) |  |  |  |  |  |
| Bile leakage |  | 3 (3.8) |  |  |  |  |  |
| Other |  | 4 (5.1) |  |  |  |  |  |
| **Surgical Complications^a^** | 79 | 18 (22.8) | 1 (33.3) | 6 (14.3) | 10 (32.3) | 1 (33.3) | .298 |
| **Non-surgical Complications ^b^** | 79 | 11 (13.9) | 0 (0) | 7 (16.7) | 4 (12.9) | 0 (0) | .738 |
| ^a^ Including bleeding, atony, anastomosis insuﬃciency, incisional hernia, wound infection, ileus, injury to  other organs, ischemia, blood transfusion  ^b^ Including DVT, PE, UTI, pneumonia, pleural eﬀusion, electrolyte disturbances  ^A^ Statistics were realised by Fisher’s exact test, Chi^2^ test, Man-Whitney *U*-Test or Kruskal-Wallis-test, as appropriate | | | | | | | |
