# Supplementary material for: Validation and applicability of the Tampa Difficulty Score for assessing procedural complexity in robotic liver surgery
Source: Surg Endosc. 2026 Feb 23;40(5):3852–62. doi: 10.1007/s00464-025-12507-5 (PMC13160962; doi:10.1007/s00464-025-12507-5)
Supplement: Supplementary file 4 — Supplementary file4 (DOCX 16 kb) [file 464_2025_12507_MOESM4_ESM.docx]

**Table 9-S:** Postoperative laboratory parameters

| **Maximum values** | **Valid cases** | **Total Cohort**  **n=79 Median [IQR] or number (%)*** | **Tampa Group 1**  **n=3**  Median [IQR] or number (%)* | **Tampa Group 2**  **n=42**  Median [IQR] or number (%)* | **Tampa Group 3 n=31**  Median [IQR] or number (%)* | **Tampa Group 4**  **n=3**  Median [IQR] or number (%)* | ***p*-value^A^** |
| --- | --- | --- | --- | --- | --- | --- | --- |
| **CRP** [mg/L] | 78 | 13.5 [6.7; 17.8] | 2.7 [1.3; 2.7] | 13.2 [7.5; 19.1] | 14.0 [7.4; 17.8] | 14.1 [3.8; 14.1] | **.037** |
| **PCT** [mg/L] | 23 | 0.33 [0.18; 0.79] | - | 0.24 [0.07; 0.33] | 0.57 [0.12; 0.79] | 0.23 [0.23; 2.16] | .621 |
| **Bilirubine** [mg/L] | 78 | 1.0 [0.6; 1.4] | 0.8 [0.4; 0.8] | 0.7 [0.5; 1.0] | 1.2 [1.0; 2.6] | 2.7 [2.0; 2.7] | **.002** |
| **ALT** [U/L] | 74 | 274 [143; 441] | 49 [46; 49] | 179 [108; 337] | 385 [262; 616] | 603 [277; 603] | **< .001** |
| **AST** [U/L] | 74 | 238 [118; 444] | 56 [40; 56] | 167 [102; 307] | 322 [203; 518] | 547 [330; 547] | **< .001** |
| **GGT** [U/L] | 78 | 138 [66; 327] | 19 [8; 19] | 89 [55; 180] | 296 [151; 586] | 262 [165; 262] | **< .001** |
| **AP** [U/L] | 73 | 136 [85; 253] | 44 [34; 44] | 107 [72; 148] | 216 [159; 284] | 192 [96; 192] | **< .001** |
| **LDH** [U/L] | 62 | 350 [283; 487] | 219 [150; 219] | 330 [237; 392] | 469 [300; 622] | 283 [283; 472] | **.003** |
| **Lactate** [mmol/L] | 73 | 1.6 [1.1; 2.7] | - | 1.2 [1.0; 1.9] | 2.4 [1.5; 3.4] | 2.8 [1.5; 2.8] | **.002** |
| ^A^ Statistics were realised by Kruskal-Wallis-test | | | | | | | |
