# Supplementary material for: Validation and applicability of the Tampa Difficulty Score for assessing procedural complexity in robotic liver surgery
Source: Surg Endosc. 2026 Feb 23;40(5):3852–62. doi: 10.1007/s00464-025-12507-5 (PMC13160962; doi:10.1007/s00464-025-12507-5)
Supplement: Supplementary file 7 — Supplementary file7 (DOCX 19 kb) [file 464_2025_12507_MOESM7_ESM.docx]

**Table 12-S:** Histopathological results

|  | **Valid cases** | **Total Cohort**  **n=79 Median [IQR] or number (%)*** | **Tampa Group 1**  **n=3**  Median [IQR] or number (%)* | **Tampa Group 2**  **n=42**  Median [IQR] or number (%)* | **Tampa Group 3 n=31**  Median [IQR] or number (%)* | **Tampa Group 4**  **n=3**  Median [IQR] or number (%)* | ***p*-value^A^** |
| --- | --- | --- | --- | --- | --- | --- | --- |
| **Entitiy** | 79 |  |  |  |  |  | **.001** |
| mCRC |  | 38 (48.1) | 0 (0) | 15 (35.7) | 22 (73.3) | 1 (33.3) |  |
| HCC |  | 7 (8.9) | 0 (0) | 4 (9.5) | 2 (6.7) | 1 (33.3) |  |
| Intrahepatic CCA |  | 11 (13.9) | 0 (0) | 8 (19.0) | 3 (10.0) | 0 (0) |  |
| Non-CRC metastasis |  | 7 (8.9) | 0 (0) | 3 (7.1) | 3 (10.0) | 1 (33.3) |  |
| Benign hepatic tumor |  | 15 (19) | 3 (100) | 12 (28.6) | 0 (0) | 0 (0) |  |
| **R-Status** | 77 |  |  |  |  |  | .803 |
| R0 |  | 69 (89.6) | 3 (100) | 37 (90.2) | 26 (86.7) | 3 (100) |  |
| R1 |  | 8 (10.3) | 0 (0) | 4 (9.8) | 4 (13.3) | 0 (0) |  |
| **Weight of resected liver tissue^a^** [g] | 77 | 292 [82; 734] | 79 [25; 79] | 180 [44; 310] | 740 [432; 106] | 514 [180; 514] | **<.001** |
| **Tumor size [cm]** | 79 | 4.1 [2.4; 4.2] | 2.4 [2.3; 2.4] | 2.9 [2.3; 5.0] | 4.5 [3.6; 5.8] | 4.8 [2.7; 4.8] | **.012** |
| *As appropriate  ^a^ Range 0.4 g - 1945 g  ^A^ Statistics were realised by Fisher’s exact test, Chi^2^ test, Man-Whitney *U*-Test or Kruskal-Wallis-test, as appropriate | | | | | | | |
