# Supplementary material for: Validation and applicability of the Tampa Difficulty Score for assessing procedural complexity in robotic liver surgery
Source: Surg Endosc. 2026 Feb 23;40(5):3852–62. doi: 10.1007/s00464-025-12507-5 (PMC13160962; doi:10.1007/s00464-025-12507-5)
Supplement: Supplementary file 8 — Supplementary file8 (DOCX 18 kb) [file 464_2025_12507_MOESM8_ESM.docx]

**Table 13-S:** Post-Hoc Liver Weight

|  | **Tampa Group 1**  **n=3**  ***p*-value^A,B^** | **Tampa Group 2**  **n=42**  ***p*-value^A,B^** | **Tampa Group 3 n=31**  ***p*-value^A,B^** | **Tampa Group 4**  **n=3**  ***p*-value^A,B^** | **d_Cohen_** | **Eta squared (η2)** |
| --- | --- | --- | --- | --- | --- | --- |
| **Tampa Group 1**  **n=3**  ***p*-value^A,B^** | n/a | 1 | **.027** | **.280** | 1.115/2.746 | .237/.653 |
| **Tampa Group 2**  **n=42**  ***p*-value^A,B^** | 1 | n/a | **<.001** | .510 | 1.313 | .301 |
| **Tampa Group 3 n=31**  ***p*-value^A,B^** | **.027** | **<.001** | n/a | 1 | 1.115/1.313 | .237/.301 |
| **Tampa Group 4**  **n=3**  ***p*-value^A,B^** | **.280** | .510 | 1 | n/a | 2.746 | .653 |
| ^A^ Statistics were realised by Independant-Samples Kruskal-Wallis-Test  ^B^ Significant Values have been adjusted by the Bonferroni-Correlation for multiple test | | | | | | |
