# Supplementary material for: Validation and applicability of the Tampa Difficulty Score for assessing procedural complexity in robotic liver surgery
Source: Surg Endosc. 2026 Feb 23;40(5):3852–62. doi: 10.1007/s00464-025-12507-5 (PMC13160962; doi:10.1007/s00464-025-12507-5)
Supplement: Supplementary file 9 — Supplementary file9 (DOCX 17 kb) [file 464_2025_12507_MOESM9_ESM.docx]

|  | **Valid cases**  **n=79** | **Total Cohort**  **n=79 Median [IQR] or number (%)*** | **Tampa Group 1**  **n=3**  Median [IQR] or number (%)* | **Tampa Group 2**  **n=42**  Median [IQR] or number (%)* | **Tampa Group 3 n=31**  Median [IQR] or number (%)* | **Tampa Group 4**  **n=3**  Median [IQR] or number (%)* | ***p*-value ^A^** |
| --- | --- | --- | --- | --- | --- | --- | --- |
| mCRC |  | 41 (51.9) | 0 (0) | 16 (38.1) | 24 (77.4) | 1 (33.3) | **< .001** |
| HCC |  | 7 (8.9) | 0 (0) | 4 (9.5) | 2 (6.5) | 1 (33.3) |  |
| Intrahepatic CCA |  | 13 (16.5) | 0 (0) | 9 (21.4) | 4 (12.9) | 0 (0) |  |
| Extrahepatic CCA |  | 2 (2.5) | 0 (0) | 1 (2.4) | 0 (0) | 1 (33.3) |  |
| Non-CRC metastasis |  | 4 (5.1) | 0 (0) | 3 (7.1) | 1 (3.2) | 0 (0) |  |
| Benign hepatic tumor |  | 12 (15.2) | 3 (100) | 9 (21.4) | 0 (0) | 0 (0) |  |
| ^A^ Statistics were realised by Fisher’s exact test, Chi^2^ test, Man-Whitney U-Test or Kruskal-Wallis-test, as appropriate | | | | | | | |

**Table 1-S:** Indications for surgery
