# Supplementary material for: Validation and applicability of the Tampa Difficulty Score for assessing procedural complexity in robotic liver surgery
Source: Surg Endosc. 2026 Feb 23;40(5):3852–62. doi: 10.1007/s00464-025-12507-5 (PMC13160962; doi:10.1007/s00464-025-12507-5)
Supplement: Supplementary file 10 — Supplementary file10 (DOCX 16 kb) [file 464_2025_12507_MOESM10_ESM.docx]

**Table 2-S:** Characteristics of surgical procedure

|  | **Valid cases** | **Total Cohort**  **n=79 Median [IQR] or number (%)*** | **Tampa Group 1**  **n=3**  Median [IQR] or number (%)* | **Tampa Group 2**  **n=42**  Median [IQR] or number (%)* | **Tampa Group 3 n=31**  Median [IQR] or number (%)* | **Tampa Group 4**  **n=3**  Median [IQR] or number (%)* | ***p*-value^A^** |
| --- | --- | --- | --- | --- | --- | --- | --- |
| **Type of surgery** | 79 |  |  |  |  |  | **< .001** |
| Minor Resection |  | 41 (51.9) | 3 (100.0) | 33 (78.6) | 5 (16.1) | 0 (0) |  |
| Major Resection |  | 38 (48.1) | 0 (0) | 9 (21.4) | 26 (83.9) | 3 (100.0) |  |
| **Intraoperative Complication** | 79 |  |  |  |  |  | .499 |
| Minor |  | 12 (15.2) | 0 (0) | 4 (9.5) | 7 (22.6) | 1 (33.3) |  |
| Major |  | 4 (5.1) | 0 (0) | 1 (2.4) | 3 (0.7) | 0 (0) |  |
| Other |  | 1 (1.3) | 0 (0) | 0 (0) | 1 (3.2) | 0 (0) |  |
| **Conversion** | 79 |  |  |  |  |  | .873 |
| None |  | 69 (87.3) | 3 (100) | 37 (88.1) | 26 (83.9) | 3 (100) |  |
| Planned |  | 9 (11.4) | 0 (0) | 5 (11.9) | 4 (12.9) | 0 (0) |  |
| Emercency |  | 1 (1.3) | 0 (0) | 0 (0) | 1 (3.2) | 0 (0) |  |
| **Blood Loss** [ml] | 79 | 200 [50; 500] | 10 [10; 10] | 100 [50; 200] | 300 [200; 600] | 500 [200; 500] | **< .001** |
| **Transfusion** | 79 | 4 (5.1) | 0 (0) | 0 (0) | 4 (12.9) | 0 (0) | .089 |
| **Peridural catheter** | 79 | 42 (53.2) | 1 (33.3) | 19 (45.2) | 20 (64.5) | 2 (66.7) | .340 |
| **Technical Parameters** | 79 |  |  |  |  |  |  |
| OR time [min] | 78 | 202 [136; 264] | 68 [54; 68] | 171 [130; 202] | 263 [234; 322] | 420 [405; 420] | **< .001** |
| **Devices** | 79 |  |  |  |  |  |  |
| Sealing |  | 58 (73.4) | 3 (100.0) | 29 (69.0) | 24 (77.4) | 2 (66.7) | .851 |
| Dissection |  | 52 (65.8) | 1 (33.3) | 25 (59.5) | 24 (77.4) | 2 (66.7) | .261 |
| Stapler |  | 48 (60.8) | 1 (33.3) | 16 (38.1) | 28 (90.3) | 3 (100.0) | **.003** |
| *As appropriate  n/a not applicable  ^A^ Statistics were realised by Fisher’s exact test, Chi^2^ test, Man-Whitney U-Test or Kruskal-Wallis-test, as appropriate | | | | | | | |
