# Supplementary material for: Validation and applicability of the Tampa Difficulty Score for assessing procedural complexity in robotic liver surgery
Source: Surg Endosc. 2026 Feb 23;40(5):3852–62. doi: 10.1007/s00464-025-12507-5 (PMC13160962; doi:10.1007/s00464-025-12507-5)
Supplement: Supplementary file 11 — Supplementary file11 (DOCX 16 kb) [file 464_2025_12507_MOESM11_ESM.docx]

**Table 3-S:** Characteristics of surgical procedures (Degree of resection)

|  | **Total Cohort**  **n=79**  **number (%)** |
| --- | --- |
| **Segmetectomy** | 18 (22.8) |
| **Bisegmentectomy** | 23 (29.1) |
| **Trisegmentectomy** | 3 (3.8) |
| **Hemihepatectomy** | 24 (30.4) |
| Left | 9 (11.4) |
| Right | 15 (19) |
| **Extended Hemihepatectomy** | 10 (12.7) |
| Left | 3 (3.8) |
| Right | 7 (8.9) |
| **Trisectorectomy** | 1 (1.3) |
